# Endométrite tuberculeuse post-ménopausique simulant un cancer de l’endomètre: à propos d’un cas

**Published:** 2012-01-15

**Authors:** Houda Elbahraoui, Abderrahman Elmazghi, Hanane Bouziane, Adil Elghanmi, Amina Lakhdar, Driss Ferhati

**Affiliations:** 1 Service de gynecologie-obstetrique, CHU Ibn Sina Rabat, Maroc; 2 Service de radiotherapie, CHU Hassan II de Fés, Maroc

**Keywords:** Tuberculose génitale, endomètre, ménopause

## Abstract

La tuberculose génitale est une affection rare dans les pays développés mais reste fréquente dans notre pays malgré la généralisation de la vaccination et la codification du traitement anti-bacillaire. La tuberculose génitale féminine est caractérisée par la fréquence des formes latentes inapparentes. Le mode de contamination est dominé par la voie hématogène provenant d’un autre foyer, avec une atteinte initiale des trompes réalisant un tableau de salpingite à partir de laquelle l’infection progresse vers les autres organes génitaux. Des cas exceptionnels sont rapportés durant la ménopause, ils sont le plus souvent secondaires à d’autres localisations extra-génitales, avec une grande latence entre le foyer initial souvent pulmonaire et la localisation génitale. Notre travail porte sur l’observation d’une patiente de 60 ans, suivie pour cancer du sein traité puis fut mise sous tamoxifène depuis 4 ans. Dans le cadre de son bilan de surveillance une échographie pelvienne de control a été réalisée, objectivant une collection intra-utérine et chez qui l’examen direct bactériologique après évacuation a objectivé une infection à *Mycobacterium tuberculosis*. Le bilan d’extension à la recherche d’autre localisation extra génitales est négatif. L’évolution sous anti-baccillaires était favorable.

## Introduction

La tuberculose et plus précisément la tuberculose génitale sévit encore à l’état endémique au Maroc, malgré les moyens mis en œuvre pour son éradication, en particulier la vaccination antituberculeuse systématique à la naissance et le traitement anti-bacillaire codifié et délivré gratuitement [1].

La tuberculose génitale féminine est caractérisée par la fréquence des formes latentes inapparentes. Le mode de contamination est dominé par la voie hématogène provenant d’un autre foyer, avec une atteinte initiale des trompes réalisant un tableau de salpingite à partir de laquelle l’infection progresse vers les autres organes génitaux.sa survenue chez une femme ménopausée est très rare [2].

Nous rapportons l’observation d’une patiente de 60 ans, traité pour cancer du sein puis fut mise sous hormonothérapie et chez qui l’échographie pelvienne de surveillance révèle une image intra-utérine faisant suspecter vue le contexte un cancer de l’endomètre.et c’est les examens histologique et bactériologique qui redressent le diagnostic et confirme son atteinte d’une endométrite tuberculeuse.

## Patient et observation

Madame A.G est une patiente de 60 ans, 2 ^e^ geste 2 ^e^ pare, ménopausée depuis 6 ans, dont le niveau socioéconomique est bas, ayant comme ATCD un cancer du sein droit pour lequel elle a bénéficié d’un traitement chirurgical conservateur suivi de 06 cycles de chimiothérapie à base de cisplatine à dose de 100mg/m^2^, doxorubicine à dose de 60mg/m^2^ (j1=j22) et d’une radiothérapie de 50 Gy sur le sein droit en totalité et un boost de 16 Gy sur le lit tumoral puis elle a été mise sous hormonothérapie depuis 4 ans. Par ailleurs on ne retrouve ni notion de contage tuberculeux, ni signes d’imprégnation tuberculeuses.

**Figure 1:**
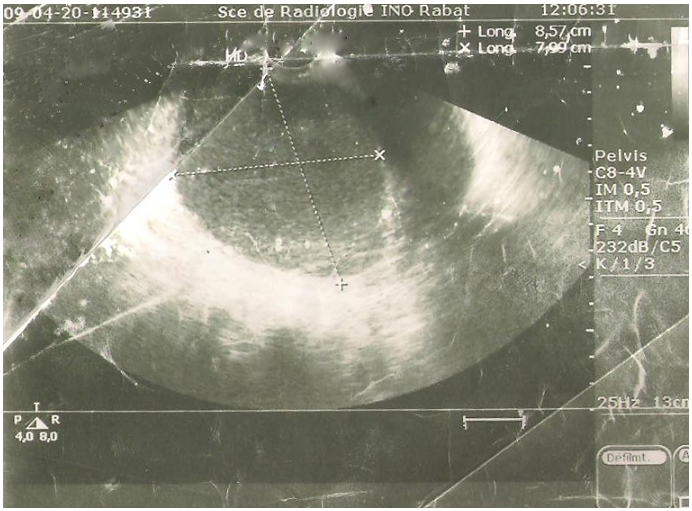
Echographie pelvienne montrant un utérus augmenté de taille, globuleux siège d’une image intra cavitaire hyperechogene homogène

Dans le cadre de son bilan de surveillance, une échographie pelvienne (Figure 1) a été réalisée, ayant montré un utérus augmenté de taille, globuleux siège d’une image intra cavitaire hyperéchogène homogène (pyométrie ?), On a suspecté initialement un processus néoplasique endométrial vue l’hormonothérapie que la patiente reçoit. L’examen gynécologique au speculum retrouve un orifice cervicale punctiforme, au toucher vaginale le col était sténosé infranchissable avec un utérus arrivant à mi-chemin de l’ombilic.

Sous sédation on a procédé à une dilatation du col utérin permettant l’issu d’un liquide épais jaunâtre caséeux, on a complété l’évacuation utérine par un curetage biopsique de l’endomètre.

L’examen anatomo-pathologique a montré la présence de granulomes epithélioides et giganto-cellulaires, avec nécroses caséeuse faisant évoquer une tuberculose caséo-folliculaire évolutives avec absence de lésions malignes.

La recherche de BAAR a été effectuée est revenue(+) et c’est la culture sur milieu spécifique de Lowenstein qui a confirmé le diagnostic par la mise en évidence du *Mycobacterium tuberculosis*.

La recherche d’un autre foyer pulmonaire ou urinaire était négative. Une triple antibiothérapie a été instaurée, associant rifampicine, isoniazide (Rifinah^®^ 300 mg) et pyrazinamide (PZA^®^ 500 mg), aux doses quotidiennes respectives de 600 et 1500 mg.

**Figure 2:**
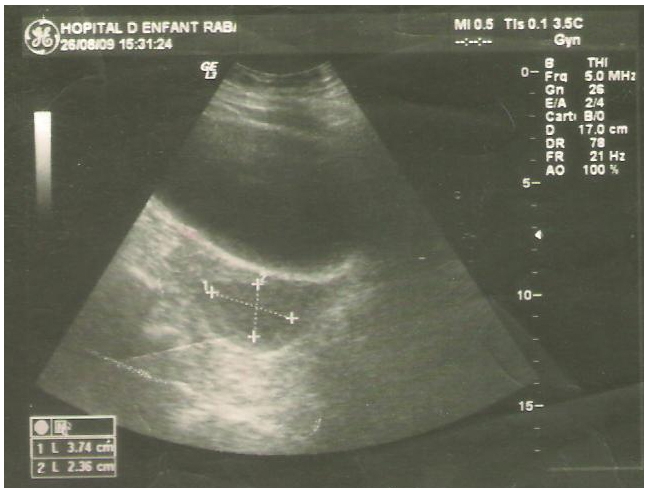
Echographie pelvienne de control 4 mois du début du traitement antibaccillaire

Elle est poursuivie pour une durée initiale de deux mois, avant le relais par une bithérapie associant rifampicine et isoniazide aux mêmes posologies.une échographie de contrôle faite après quatre mois de traitement a montré une nette régression de la collection (Figure 2). La durée totale du traitement a été fixée à six mois avec une bonne évolution clinique.

## Discussion

La tuberculose (TB) est toujours d’actualité. Au niveau mondial neuf millions de nouveaux cas et un million et demi de décès sont enregistrés annuellement. La tuberculose extra-pulmonaire représente une proportion progressivement plus grande de nouveaux cas dans les pays développés, et cette tendance augmente toujours. La réapparition de la TB dans le monde développé peut être expliquée par un grand nombre d′immigrés des payés endémique [3,4], et par l’infection par le VIH (virus de l’immunodéficience humaine) qui est un facteur important conduisant à la réapparition globale de la tuberculose, cependant La pandémie de VIH pose des problèmes majeurs pour le programme de gestion de tuberculose. [5,6].

Au Maroc, durant l’année 2007, 500 à 1.000 décès dus à la tuberculose, et 25.562 nouveaux cas ont été répertoriés, soit une stagnation de l’incidence de la maladie [1].

L′incidence vraie de la tuberculose génitale n′est pas connue étant donné que beaucoup de cas restent non diagnostiquée. Son diagnostic est classiquement porté chez une femme en période d’activité génitale. Mais cette pathologie peut se voir à tout âge, aussi bien chez les femmes ménopausées que chez les jeunes filles en période pré-pubertaire, où les synéchies sont responsables d’une aménorrhée primaire [7].

Des cas exceptionnels de tuberculose génitale sont rapportés durant la ménopause, ils sont le plus souvent secondaires à d’autres localisations extra-génitales, avec une grande latence entre le foyer initial souvent pulmonaire et la localisation génitale [2].

La tuberculose génitale féminine est caractérisée par la fréquence des formes latentes, inapparentes. Le mode de contamination est dominé par la voie hématogène provenant d’un autre foyer, La transmission directe entre les partenaires sexuels a été documentée. La diffusion d′autres foyers intra péritonéaux est rare. Les trompes utérines sont affectées dans presque 100% des cas, suivis de l′endomètre dans 50%, les ovaires dans 20%, le col utérin dans 5%, et le vagin et la vulve dans 8].

L′endométrite tuberculeuse chez les jeunes femmes est presque toujours associée à la salpingite tuberculeuse contrairement aux femmes ménopausées où souvent l’endométrite est isolée sans atteinte tubaire [3].

La tuberculose génitale est rare chez les femmes ménopausée et responsable de 1% de métrorragies post ménopausique. L′endomètre est affecté dans 60-70%. Il est difficile d’expliquer l′incidence limitée de cette maladie chez cette catégorie d’âge. La plupart des auteurs croient qu′un endomètre atrophique représente un milieu pauvre pour la croissance de la mycobactérie [2,3].

La TB génitale est souvent asymptomatique ainsi la maladie peut ne pas se manifester qu’après des années de la primo-infection. Les motifs de consultation les plus rapportées étaient l′infertilité (44%), la douleur pelvienne (25%), le saignement vaginal (18%), l′aménorrhée (5%), la leucorrhée (4%), et les métrorragies post-ménopausiques (2%). Rarement une masse abdominale, une ascite, ou un abcès tubo-ovarian [9].

Le bilan biologique est d′un intérêt médiocre : vitesse de sédimentation accélérée, lymphocytose, modifications des gammaglobulines.

La radiographie pulmonaire peut montrer des séquelles parenchymateuses ou pleurales, moins souvent des lésions évolutives. L′urographie intraveineuse est utile en raison de la fréquence de la tuberculose urinaire associée.

Les techniques d’imagerie ne sont pas spécifiques. L’hystérosalpingographie peut montrer des adénopathies pelviennes calcifiées, et des synéchies de la cavité utérine, réalisant un aspect typiquement en doigt de gant ou, si l’utérus est entièrement symphysé, l’opacification de l’endocol ou de l’isthme seulement. On peut voir également des sténoses des trompes leur donnant un aspect rigide, des images d’abcès ou d’hydrosalpinx non-caractéristiques [8].

Le diagnostic de certitude est obtenu par la mise en évidence du *Mycobacterium tuberculosis*, soit à l’examen direct microscopique, soit après mise en culture de prélèvements pathologiques. Le matériel analysé est obtenu par curetage biopsique endométrial, ou par laparoscopie voire laparotomie avec parfois hystérectomie [8].

Chez notre patiente, la première hypothèse diagnostique compte tenu de l’âge, l’antécédent de cancer du sein, le traitement hormonal qu’elle a reçu pendant 4 ans et des signes cliniques, était un processus néoplasique endométrial. La revue de la littérature retrouve d’ailleurs quelques cas d’association d’une tuberculose de l’endomètre et d’une néoplasie justifiant d’une surveillance rapprochée pendant et après le traitement anti-tuberculeux.

La prise en charge thérapeutique standard est actuellement bien codifiée et repose sur l’administration quotidienne pendant 6 mois d’Isoniazide et de Rifampicine, associée pendant les 2 premiers mois de Pyrazinamide et Éthambutol. La surveillance clinique et paraclinique s’effectue régulièrement tout au long du traitement [8].

Le traitement chirurgical est indiqué en cas de : persistance de masses annexielles malgré le traitement médical, en particulier l′abcès froid; rechute de la tuberculose endométrial après une année de traitement; persistance des douleurs pelviennes après 3 mois de traitement ou lorsqu′elles n′ont pas totalement disparu au terme d′un an de traitement; métrorragies persistant après guérison anatomique et clinique; fistules qui ne se tarissent pas [10].

La chirurgie devrait être réalisé au moins 6 semaines après le début du traitement antibaccillaire car il réduit le risque des complications per opératoires et facilite l’abord chirurgical [3].

Notre patiente a bénéficié de 6 mois de traitement médical par les antibaccillaire avec bonne évolution clinique, chez elle il y avait pas d’indication chirurgicale.

## Conclusion

La tuberculose reste fréquente mais s’exprime rarement par une atteinte génitale. Ce sont surtout des femmes jeunes de bas niveau socio-économique, consultant devant une stérilité. Il faut cependant savoir l’évoquer devant une symptomatologie pelvienne traînante, quelque soit l’âge, et réaliser facilement des examens cytobactériologiques.
